# The effects of *Bidens alba* invasion on soil bacterial communities across different coastal ecosystem land-use types in southern China

**DOI:** 10.1371/journal.pone.0238478

**Published:** 2020-10-28

**Authors:** Yue Wang, Juyu Lian, Hao Shen, Yunlong Ni, Ruyun Zhang, Yun Guo, Wanhui Ye

**Affiliations:** 1 Key Laboratory of Vegetation Restoration and Management of Degraded Ecosystems, South China Botanical Garden, Chinese Academy of Sciences, Guangzhou, Guangdong, China; 2 Center of Plant Ecology, Core Botanical Gardens, Chinese Academy of Sciences, Guangzhou, Guangdong, China; 3 Southern Marine Science and Engineering Guangdong Laboratory (Guangzhou), Guangzhou, China; 4 Guangdong Provincial Key Laboratory of Applied Botany, South China Botanical Garden, Chinese Academy of Sciences, Guangzhou, China; 5 University of Chinese Academy of Sciences, Beijing, China; Shandong University, CHINA

## Abstract

Environments in both biotic and abiotic ecosystems have been affected by the colonization of non-native flora. In this study, we examined the effect of *Bidens alba* invasion on different land-use types along a coastline in southern China. Bacterial communities in each site were determined using 16S rDNA sequencing, and soil physicochemical properties were analyzed using standard methods. Although our results indicated that *B*. *alba* invasion did not have a significant effect on the alpha diversity of bacteria, it caused significant differences in soil bacterial community composition between invaded and uninvaded soil across different land-use types. Beta diversity and several physicochemical properties in forest, orchard and waterfront environments were recorded to be more susceptible to *B*. *alba* invasion. A high proportion of the variation of bacterial communities can be explained by a combination of environmental variables, indicating that environmental selection rather than plant invasion is a more effective process in coastal microbial assemblages. By comparing topological roles of shared OTUs among invaded and uninvaded soil, keystone taxa in invaded soil were identified. Acidobacteria was the major phyla involved in the invasive process which could be driven by environmental selection. How key phyla react in our experiment should be verified by further studies.

## Introduction

In conjunction with increasing levels of atmospheric CO_2_, anthropogenic land-use change and pollution, plant invasion is a key driver of ongoing global change and a major threat to biodiversity [1–3]. Plant invasions can potentially alter the structure and function of recipient ecosystems, especially in coastal ecosystems [4, 5]. Compared to native plants, invasive plants are characterized as having higher net primary productivity and litter input, thereby reducing growth, fitness and abundance of native species, as well as decreasing diversity of native plant communities [6, 7]. Due to plants such as *Alnus trabeculosa* and *Rosa rugosa* having a rapid effect on local microbial communities, soil affected by invasive species may record an increase in soil bacterial diversity and soil nutrients [8, 9]. Invasive plants can also have selective accumulated specific bacteria, such as soil N-fixing bacterial communities, thereby facilitating invasive processes [10–13].

Significant changes in ecosystems due to changing land-use related to urban development have been recorded, resulting in an alteration of the microclimate associated with changing temperature, humidity and light regimes [14]. More importantly, changes in land-use type can significantly boost invasion in regional areas and enhance the risk of invasion by non-native plants [15–17]. One notable reason for these changes is related to invasive plants rapidly using and changing local resources, thereby gaining favorable habitats via plant-soil feedback compared to native species [18, 19]. It is therefore important to increase our understanding regarding the impact of invasive plants on microorganisms caused by changing land-use types.

*Bidens alba* (formerly *B*. *pilosa* L.) is an invasive plant species mainly occurring in the subtropics and tropics; this species is recorded to have an extensive range in central and southern China [20]. Previous studies have shown that *B*. *alba* invasion can modify soil microbial community composition, probably creating a favorable soil environment for self-benefit [21–23]. *B*. *alba* mainly presets in agricultural and disturbed areas through strong mutualism by a unique soil-plant feedback. Although invasion processes and control strategies have been examined [23, 24], interactions between invasion plants and soil microbes encountered in different land-use types are not fully understood. Due to the size of microorganisms and their high dispersal capacity, they generally connect with each other to develop complex networks within an ecological niche [25]. Exploring co-occurrence patterns between soil microorganisms can help to identify potential biotic interactions and habitat affinities [26]. Co-occurrence networks have been used to examine broad medical and ecological consequences, such as forest management, immunological processes in gut microbiota, agriculture practices and precipitation changes [27–31]. Co-occurrence networks with structural equation modeling were used by Mamet et al. [32] to predict the evolution of microbial networks and keystone OTUs during smooth brome (*Bromus inermis*) invasion in Canada. However, the responses of microbial networks to the invasion of alien species are still unclear. Therefore, comparing bacterial networks between invaded and uninvaded soil is important to advance research on keystone taxa.

In our study, physicochemical properties and soil bacterial community responses (i.e., composition, function and the bacterial network) to *B*. *alba* invasion in coastal ecosystems are examined, as well as associations with above-ground vegetation diversity. We hypothesize that: 1) physicochemical properties and soil bacterial diversity will vary with *B*. *alba* invasion among different land-use types; and 2) interactions between bacteria members will increase during the invasion process.

## Materials and methods

### Study design

**Fig 1:**
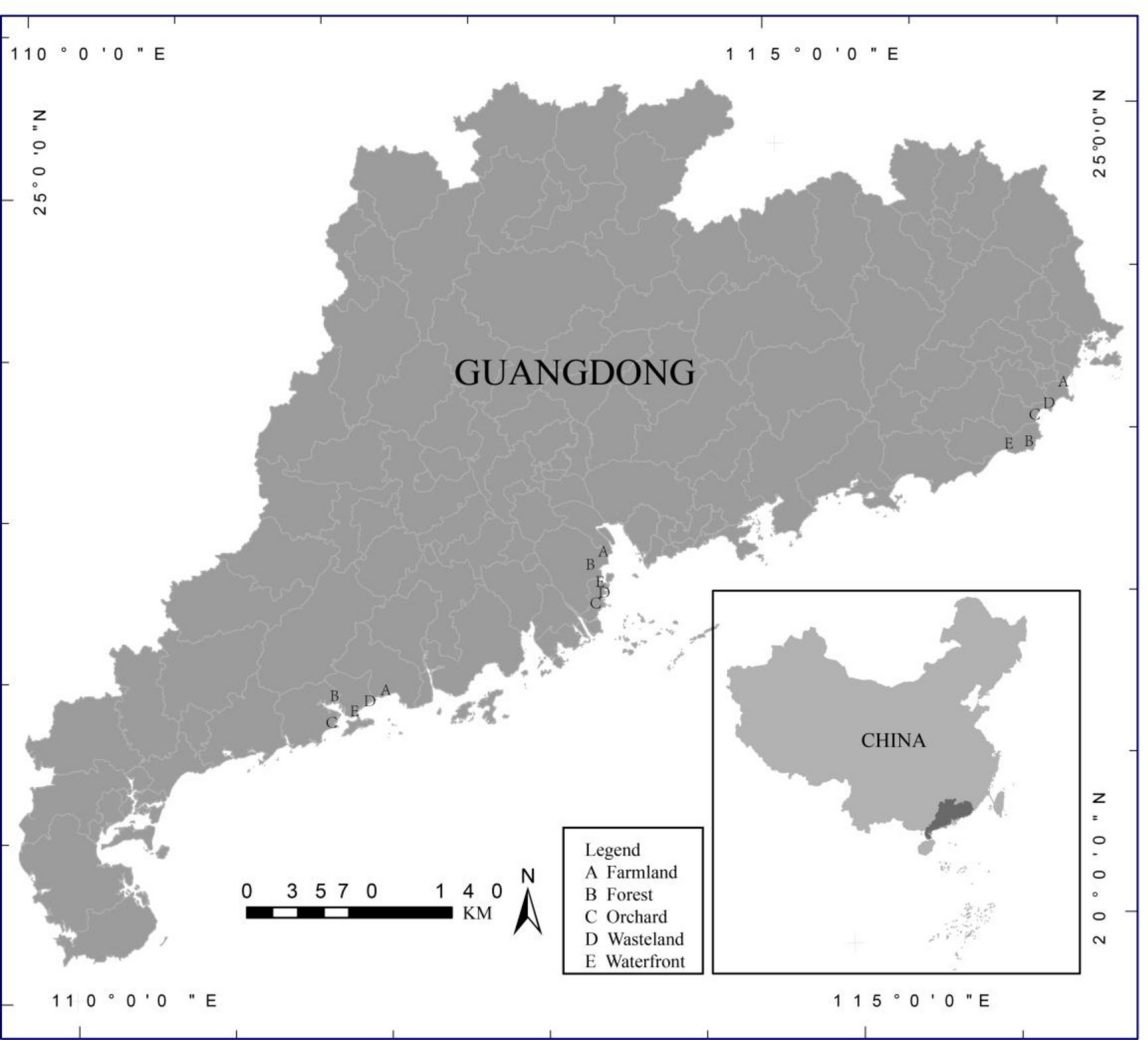
Location of the study area in Guangdong Province and 15 study sites.

Sampling was undertaken along a 5 km of coastline in south Guangdong, China, in October 2018. The total length of coastline in Guangdong Province is 4114 km. Annual mean temperature in the study region is 21.2–23.3°C, annual mean sunshine hours is 1730–2320 hrs, and annual mean precipitation is 1341.0–2382.8 mm. In order to examine the effects of *B*. *alba* invasion, the coastline ecosystem was divided into three study regions: east, central and west coastline. Land-use type along the coastal areas was classified as farmland, forest, orchard, wasteland and waterfront. Each land-use type was examined in triplicate in the three study regions (Fig 1). Soil samples 1 × 1 m (in triplicate) were collected from quadrats located in each land-use type from invaded and uninvaded soil (CK). Soil samples were homogenized from five cores (samples from the top 10 cm) randomly collected in each sample site. In total, 90 soil samples were collected for this study. The number of individuals of all plant species were calculated in all quadrats. The relative abundance of *B*. *alba* in the invaded soil quadrats was calculated as the ratio of the number of *B*. *alba* individuals to the number of individuals of all plant species. Soil collected from the study sites were classified as latosolic red soil and yellow soil [33]. All soil samples were kept in sealed bags and stored at -20°C prior to analysis.

### Ethics statement

This study was undertaken along a 5 km of coastline in south Guangdong Province, China. Land-use type was classified as farmland, forest, orchard, wasteland and waterfront. Permission to conduct the study on this site had been given by the owners of the farmland and orchard. The forest samplings are not in conservation area. No specific permissions were required for wasteland and waterfront. We confirmed that no endangered or protected species were involved in all field studies.

### Soil physicochemical properties and plant community diversity

Soil pH and electrical conductivity (EC) were measured *in situ* using a digital soil acidity meter and a digital soil EC tester. Standard methods were adopted to measure soil physicochemical properties. Total organic carbon (TOC) was measured using potassium dichromate heating oxidation-volumetric method, total nitrogen (TN) was measured using the Kjeldahl method, available nitrogen (AN) was measured using the alkali-hydrolyzed distilling method, total phosphorus (TP) was measured using NaOH digestion Mo-Sb colorimetry methods, available phosphorus (AP) was measured using the Olsen method, total potassium (TK) and available potassium (AK) were extracted with HF-HNO3-HClO4 and ammonium acetate, respectively, and determined using flame photometry. Invaded and uninvaded plots were compared using t-test for dependent samples. Shannon's diversity index (H’ = ∑PilnPi), relative abundance of *B*. *alba* and relative richness were used to calculate plant community diversity in each plot. Principal coordinate of neighbor matrices (PCNM) vectors with the PCNM package was used to derive spatial variables from geographic coordinates (latitude and longitude) [34, 35]. PCNM can be applied to any set of sites providing a good coverage of the geographic sampling area. Forward-selected procedures were used to select subsets of above-ground plants, soil physicochemical properties and PCNM variables in the PACKFOR package. These variables were then subjected to variation partitioning using the VEGAN package. Variation partitioning could assess the amounts of bacteria variation explained together by the environment and space, or by one component whilst the other component is controlled [36]. In order to determine the proportion of variation in bacterial communities, variation partitioning was implemented with adjusted R^2^ values [37].

Soil DNA extraction and PCR amplification of microbial DNA was extracted using HiPure Soil DNA Kits according to the manufacturer’s protocols. The 16S rDNA V3-V4 region of the ribosomal RNA gene were amplified by PCR, with 95°C for 2 min, followed by 27 cycles at 98°C for 10 s, 62°C for 30 s, and 68°C for 30 s, with a final extension at 68°C for 10 min. During this process, 341F: CCTACGGGNGGCWGCAG and 806R: GGACTACHVGGGTATCTAAT primers were used, where the barcode was an eight-base sequence unique to each sample. PCR reactions were performed in triplicate in a 50 μL mixture containing 5 μL of 10 × KOD Buffer, 5 μL of 2.5 mM dNTPs, 1.5 μL of each primer (5 μM), 1 μL of KOD Polymerase, and 100 ng of template DNA.

### Co-occurrence network analysis

Co-occurrence network analysis was inferred based on the Molecular Ecological Network instruction (http://ieg4.rccc.ou.edu/mena/) [38, 39]. In order to reduce rare OTUs, we removed OTUs with relative abundances less than 0.1% of the total number of bacteria sequences. Using the Random Matrix Theory (RMT) approach, the ecological network was constructed using the following steps: Firstly, we standardized the distribution matrix of invaded and uninvaded bacteria into the relative abundance for subsequent Pearson correlation analysis and network construction. OTUs that appeared in less than half of all samples were excluded. Secondly, an appropriate threshold (similarity threshold) for defining network structure was defined to obtain an adjacency matrix, thereby encoding the strength of the connection between each pair of nodes. Finally, network properties were calculated within “global network properties”, “individual nodes centrality” and “module separation and modularity calculation”. Individual node properties in the network were described using the following topological features: average degree (also termed connectivity), which describes the topological property of a node in a network; average clustering coefficient, which evaluates the tendency of neighbors of a node to connect with each other; average geodesic distance, which represents the shortest path length between the connections of any two nodes; module, where a group of nodes are connected more densely to each other than to other nodes; and modularity, which demonstrates a network which could be naturally divided into communities or modules, calculated using Newman’s method [40].

Modules were detected using the greedy modularity optimization method, and connectivity was determined based on within module connectivity (Zi) and among module connectivity (Pi) of each node. Common network properties in complex community systems include scale-free, small-world and hierarchy [41]. According to the simplified criteria, all species can be divided into four groups: peripherals (Zi <2.5 and Pi <0.62), connectors (Pi >0.62), module hubs (Zi >2.5) and network hubs (Zi >2.5 and Pi >0.62). From an ecological perspective, peripherals can be considered as specialists (always linked to nodes within their own modules) while module hubs and connectors are suggested as generalists (highly connected to numerous nodes in their own modules or to other modules) and network hubs as super generalists (acting as both module hub and connector) [42].

## Results

### Relationships between bacterial community and soil chemical properties

**Fig 2:**
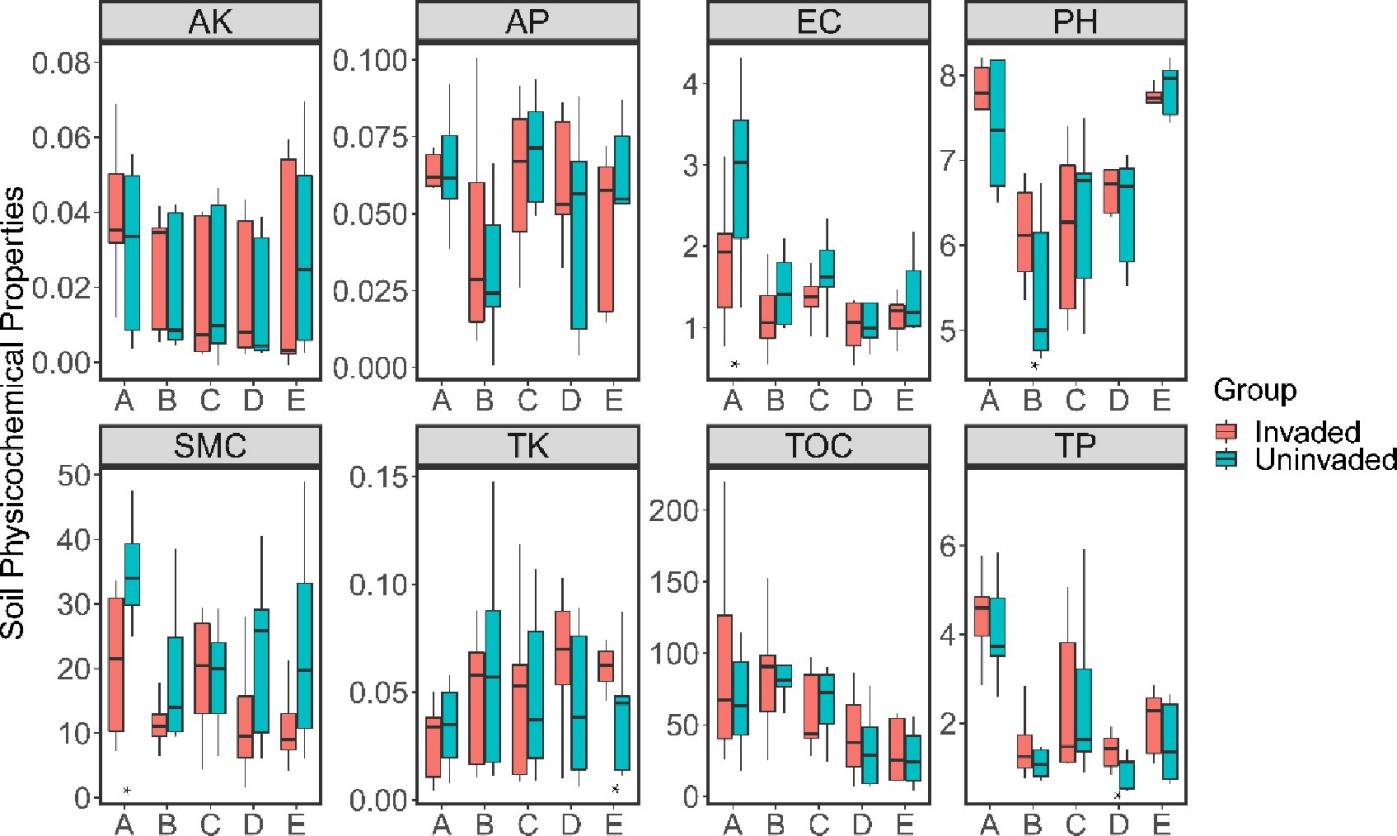
Difference of *B. alba* invasion on physicochemical properties. A: farmland, B: forest, C: orchard, D: wasteland, E: waterfront. Red box represent invaded soil, blue box represent uninvaded soil. Invaded and uninvaded soil were compared using t-test. Significant differences are marked with asterisks. *: p<0.05.

Physicochemical results (Fig 2) indicated that *B*. *alba* invasion was associated with changes in soil physicochemical properties in the different land-use types, notably pH in forest soil (t = 2.1, df = 16, *p* = 0.05), soil moisture content in farmland soil (t = -2.3, df = 16, *p* = 0.03), soil electrical conductivity in farmland soil (t = -2.5, df = 16, *p* = 0.02), total phosphorous in wasteland soil (t = 2.8, df = 16, *p* = 0.01) and total potassium in waterfront soil (t = 1.8, df = 16, *p* = 0.08). Land-use type was negatively correlated with bacteria alpha diversity whilst soil pH and soil EC were positively correlated with microbial phylogenetic diversity. Soil available potassium was negatively correlated with Shannon and phylogenetic diversity, and soil available nitrogen recorded a positive correlation (Table 1).

**Table 1:** Spearman’s rank correlation analysis between soil properties, above-ground diversity, land-use type and bacteria alpha diversity.

	Shannon index		Phylogenetic diversity	
	Coefficient	P value	Coefficient	P value
**Type**	-0.238	0.024^b^	-0.202	0.057
**Invade**	-0.018	0.865	0.055	0.603
**PH**	0.188	0.076	0.230	0.029^b^
**SMC**	-0.002	0.982	0.125	0.239
**EC**	0.021	0.843	0.216	0.041^b^
**TOC**	0.305	0.003^c^	0.195	0.066
**TN**	0.433	<0.001^d^	0.277	0.008
**C/N**	-0.012	0.909	0.058	0.589
**TP**	0.332	0.001^c^	0.284	0.007
**N/P**	0.032	0.763	-0.127	0.232
**AP**	-0.071	0.506	0.042	0.693
**AN**	0.224	0.033^b^	0.429	<0.001^d^
**TK**	0.085	0.426	-0.181	0.087
**AK**	-0.247	0.018^b^	-0.509	0.000^d^
**Above Shannon**	0.004	0.970	0.009	0.936
**Above Simpson**	0.004	0.971	0.024	0.823
**Above RA**	0.016	0.884	-0.072	0.502
**Jsw**	0.052	0.636	0.085	0.441

All the P values were adjusted with FDR method.

b:p<0.05.

c:p<0.01.

d:p<0.001

**Fig 3:**
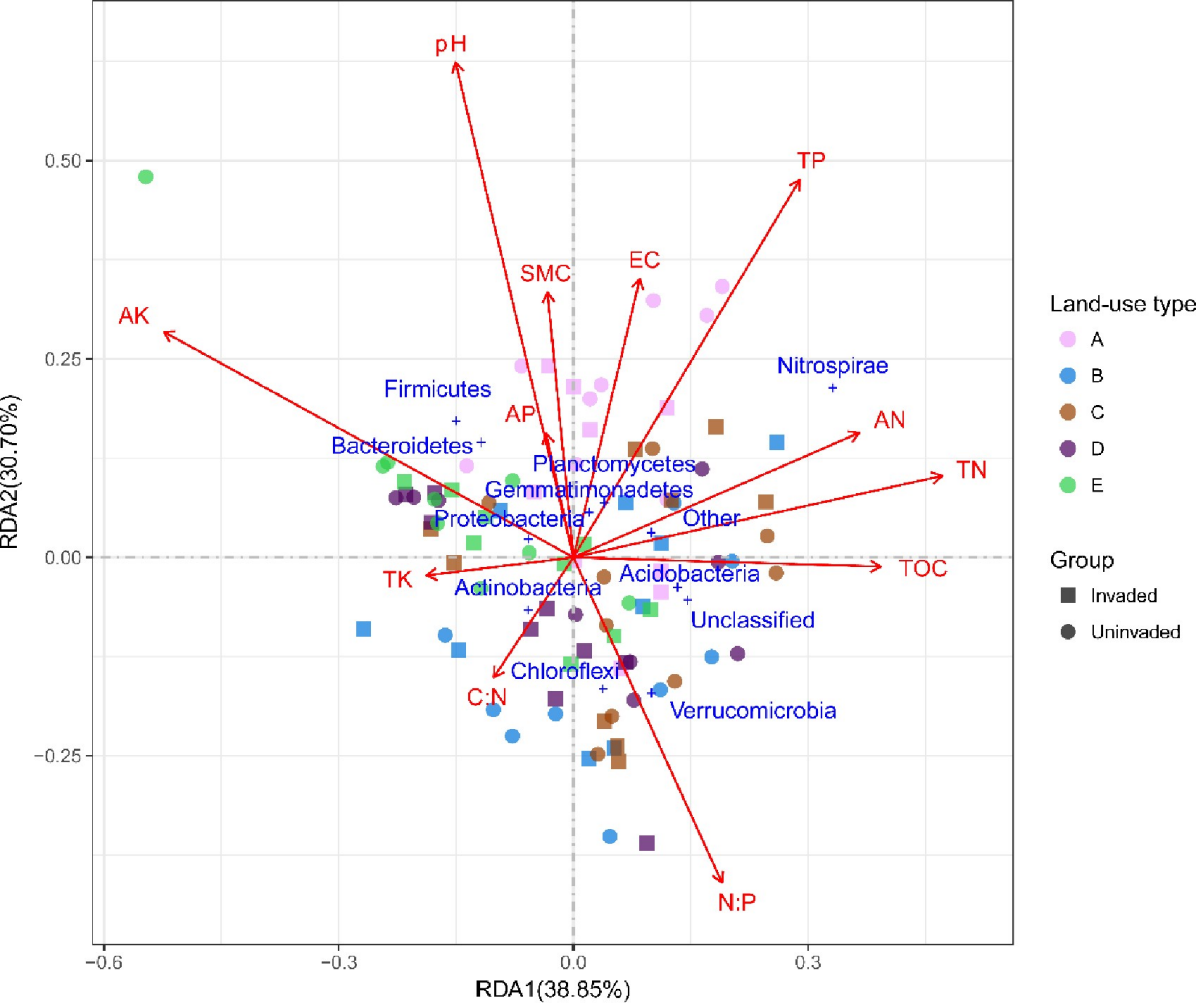
Redundancy analysis (RDA) identified seven selected environmental variables and two diversity index shaping the bacterial communities. A, farmland; B, forest; C, orchard; D, wasteland; E, waterfront. EC, electrical conductivity; TOC, total organic matter; TP, total phosphorus; TK, total potassium; AK, available potassium; TN, total nitrogen.

Factors influencing bacterial community composition in the coastline soil were identified using redundancy analysis (RDA) (Fig 3). The first component (RDA1) explained 38.8% of variation and the second component (RDA2) only explained 30.7% of variation. Based on these results and vector length, the most important soil chemical environmental variable was pH (R^2^ = 0.62, *p*<0.001), with AK (R^2^ = 0.5, *p*<0.001) and N: P (R^2^ = 0.43, *p*<0.001) also recording a strong effect on driving the microbiome in coastline soil. Total phosphorus and soil pH recorded a negative correlation with the majority of the dominant phyla, and other soil properties recorded a positive correlation with bacteria abundance. For example, a higher abundance of Gemmatimonadetes and Planctomycetes recorded a strong positive correlation with the Shannon’s diversity of above-ground vegetation and TOC. In contrast, only Actinobacteria abundance recorded a strong positive correlation with the relative abundance of *B*. *alba* and pH.

### Diversity and composition of soil bacteria community

**Fig 4:**
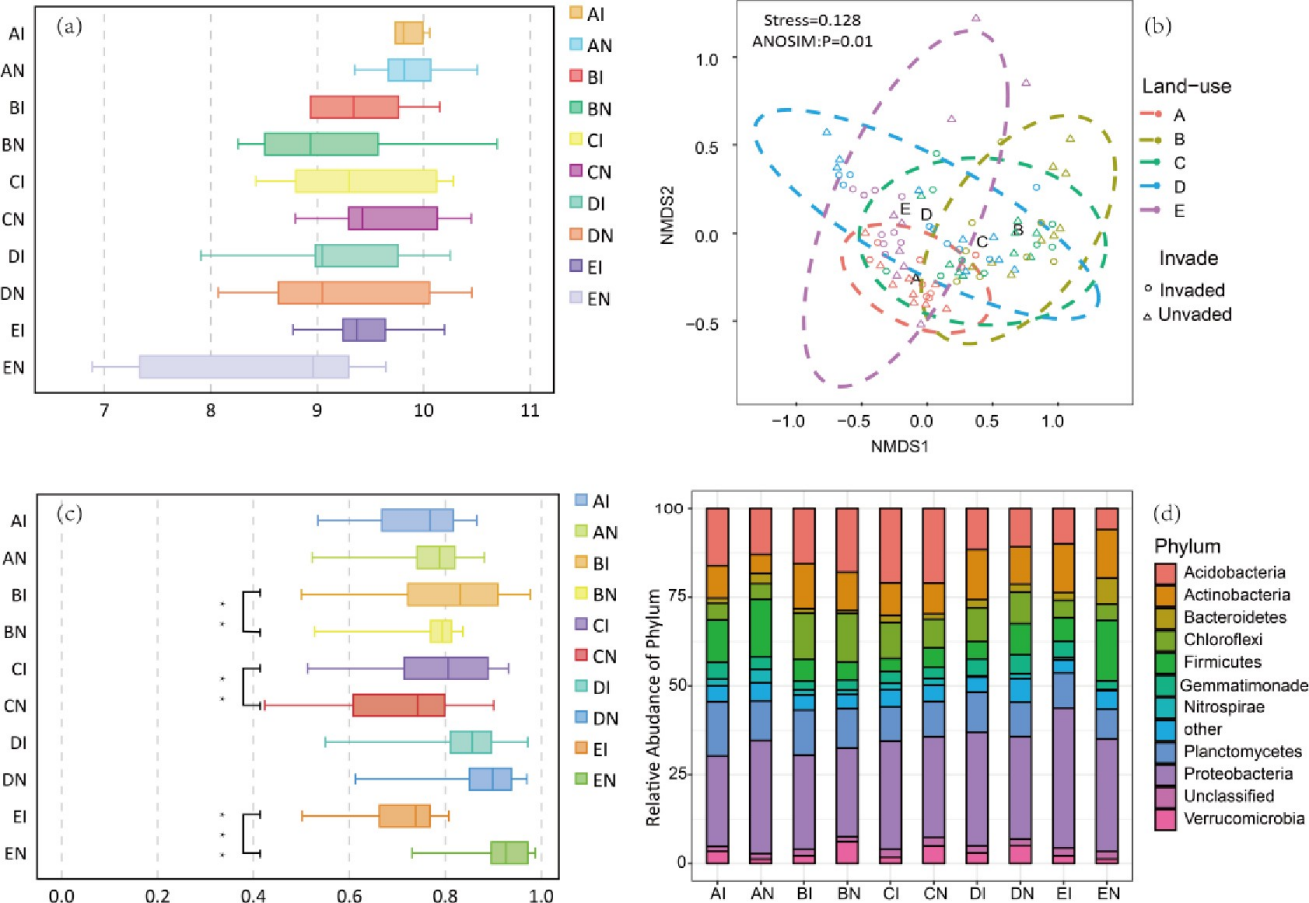
(a) Shannon-Weiner Index of bacteria community; (b) Bray-Curtis similarity of the bacteria community structure as indicated by Non-metric multidimensional scaling analysis (NMDS); (c) Bray-Curtis similarity of the bacteria community structure, Wilcoxon Rank test were used to invaded and uninvaded pairs, ** and *** indicate significant differences at 0.05 and 0.01 probability level; (d) Bacterial communities composition in invaded and uninvaded soil of five land-use type. A, farmland; B, forest; C, orchard; D, wasteland; E, waterfront. I represent invaded soil; N represent uninvaded soil.

Relationships between sample size and OTUs numbers, identified using a rarefaction curve (S1 Fig), indicated that OTUs numbers sharply increased with sample size. No significant difference in alpha diversity was recorded between invaded and uninvaded soil in all five land-use types (Fig 4). In contrast, *B*. *alba* invasion significantly decreased beta diversity in the forest and orchard land-use types; results for the waterfront land-use type were opposite. According to NMDS results, the composition of the bacteria community significantly differed among the five land-use types (PERMANOVA test: R^2^ = 0.189, *p* = 0.001). Bacterial communities were dominated by Proteobacteria, Acidobacteria, Planctomycetes and Actinobacteria, recorded as being common bacterial phyla in soils globally. Excluding unidentified sequences, a total of six phyla were recorded, accounting for 84% of total sequences. Invasion therefore increased the relative abundance of Actinobacteria in all five land-use types. The change of main bacteria was most obvious in the waterfront land-use, especially for Firmicutes which increased to 17% in uninvaded soil.

### Variation partitioning of bacterial community

**Fig 5:**
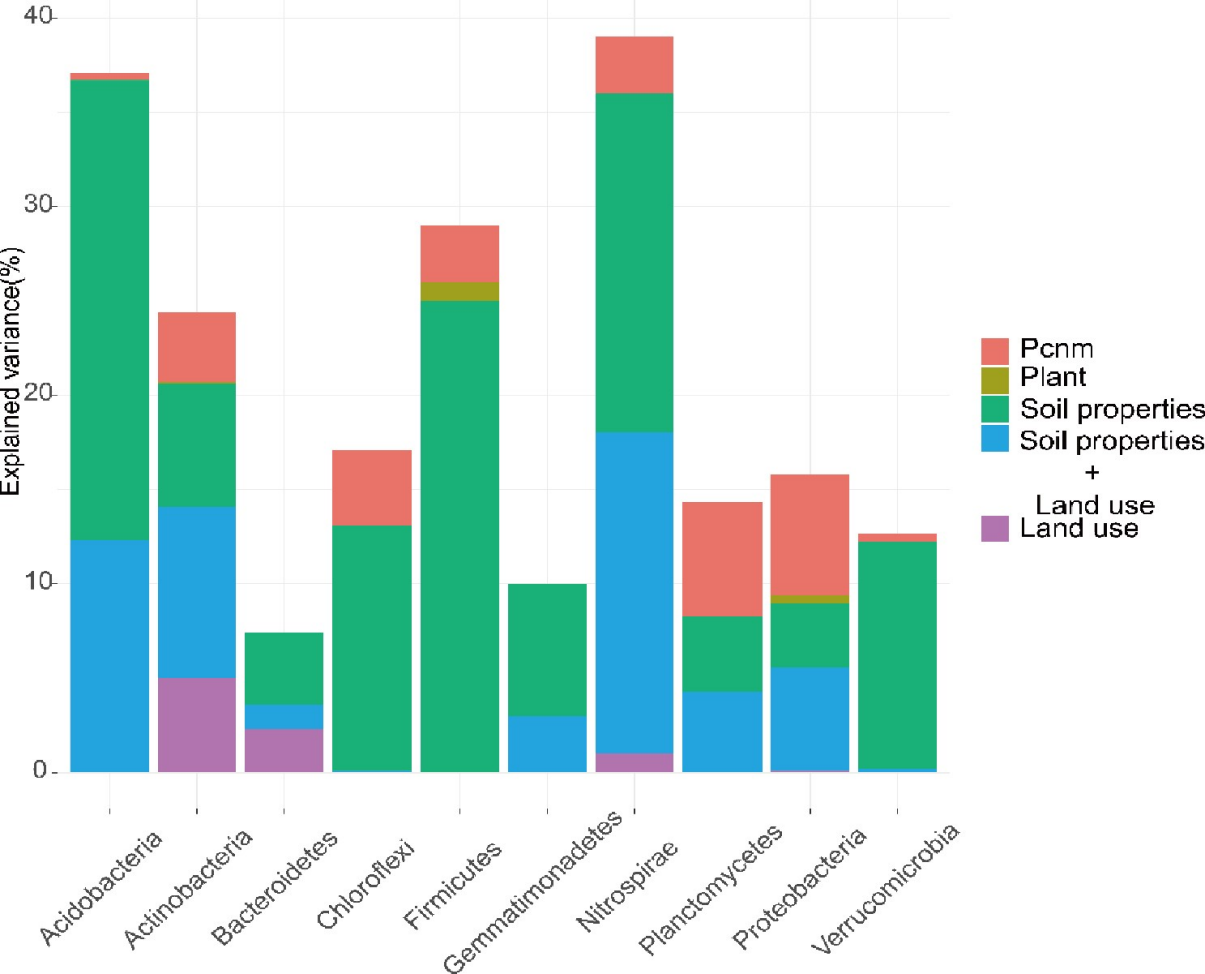
Variance partitioning of the microbial phyla across coastal zone according to environmental and spatial parameters. The top 10 microbial phyla are ranked from the most to the least abundant. The explained variance corresponds to the sum of the adjusted R2 values of the significant parameters within the contextual groups (soil properties, land-use type, spatial descriptors, above-ground plants, interactions between soil properties and land use type). The threshold for statistical significance was set at 0.01. Missing values indicate that no variable of the related group was retained in the model.

Variance partitioning analysis was undertaken to quantify the contribution of spatial distance (PCNM variables), above-ground plants, soil properties and land-use to bacterial community variation. By using a forward selection procedure, five environmental variables (pH, TOC, EC, TK and AK), three PCNM variables and the relative abundance of *B*. *alba* were selected as explanatory variables. All variables explained 32% of the variation of bacterial communities (S2 Fig), with the pure effect of plant variables accounting for 1.1% of variation in the bacterial community. The effect of total environment (soil properties and land-use type) variables explained 24% of variation of the bacterial community. In contrast, the total explained variance in phyla ranged from 7.4% (Bacteroidetes) to 39% (Nitrospirae). For 10 phyla, the influence of selection by soil properties, spatial distance and land-use type were stronger than the relative abundance of invasive plants. Soil properties were the main factors in the process of selection for Acidobacteria (24.4%) and Firmicutes (25%), and land-use type was the main factor for Actinobacteria (5%). Based on phylum results (Fig 5), interactions between soil properties and land-use type explained between 0% and 17% (Nitrospirae) of total variance.

### Co-occurrence network analysis

**Fig 6:**
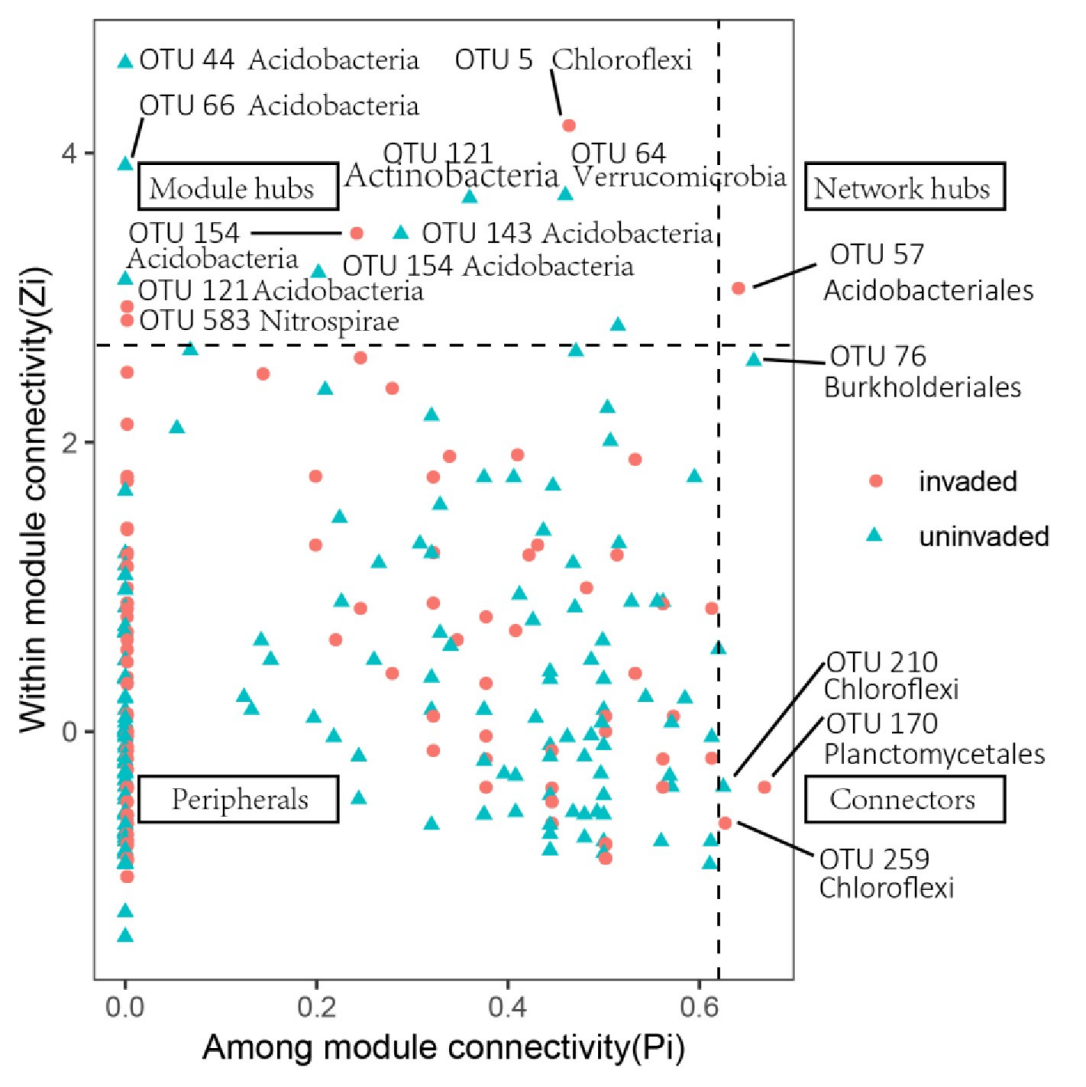
ZiPi-plot showing distribution of OTUs based on their module-based topological roles. Red dots represents invaded soil, blue dots represents uninvaded soil.

As highlighted by Barberan et al. [26], co-occurrence network analysis may provide insights into the structure of microbial communities positively or negatively correlated due to niche overlap, niche partitioning, phylogenetic similarity, mutualistic relationships or resource competition. In order to estimate bacteria coexistence under different land-use types, co-occurrence networks were established for both invaded and uninvaded soil by *B*. *alba*. Distinct features of the network organization and different interactions among bacteria members were highlighted by the topological properties of the networks varying persistently among uninvaded and invaded soil. In summary, two networks fitted a power-law (R^2^ = 0.913–0.932) (Table 2, S3 Fig). Networks in invaded and uninvaded soil had 260 and 313 total nodes, respectively. The number of total links in uninvaded soil was 266% higher than that in invaded soil. The average connectivity (avgK) was higher in uninvaded soil (7.335) while it decreased in invaded soil (3.315), which indicating high numbers of neighbors per node in the uninvaded soil network. A network hub is important to the coherence of both the network and its own module [42]. In our study, the most prominent keystone taxa in invaded networks were members of OTU 57 (phylum: Acidobacteria; class: Acidobacteria; order: Acidobacteriales), two nodes were identified as connectors in the invaded network (OTU 170 and OTU 259, Acidobacteria and Chloroflexi phylum, respectively), and one node was identified as a connector in the uninvaded network (OTU 210; phylum: Firmicutes, class: Bacilli, order: Bacillales) (Fig 6).

**Table 2:** Main properties of bacterial networks in invaded and uninvaded soil.

		Invaded	Uninvaded
**Empirical networks**	Similarity threshold (St)	0.74	0.74
	Total nodes	260	313
	Total links	431	1148
	number of module	30	27
	Modularity	0.7	0.426
	R square of power-law	0.932	0.913
	Average clustering coefficient (avgCC)	0.174	0.322
	Average connectivity (avgK)	3.315	7.335
	Average path distance (GD)	5.495	3.501
**Random networks**	Average clustering coefficient (avgCC)	0.028 +/- 0.006	0.153 +/- 0.008
	Average path distance (GD)	3.971 +/- 0.073	2.970 +/- 0.030
	Modularity	0.552 +/- 0.007	0.286 +/- 0.005

Both the properties from Empirical networks and Random networks were presented.

## Discussion

The effects of *B*. *alba* invasion on soil bacterial community composition and physicochemical properties have been widely discussed [23, 43]. Although invasion of *B*. *alba* did not significantly affect bacterial α diversity, obvious differences in soil bacterial community composition were recorded between invaded and uninvaded areas across different land-use types in southern China. This finding supported our first hypothesis. Specifically, a higher relative abundance of Actinobacteria, Firmicutes and Bacteroidetes were observed in invaded soil compared with uninvaded soil (Fig 2). This bacterial community composition may be explained by the different functions of microorganisms. For example, Actinobacteria and Bacteroidetes can successfully colonize new environments by dispersal via aerosolized soil dust [44]. Resistant physiological stages have also been formed by Firmicutes and Actinobacteria to allow them to survive in hostile environments [45, 46]. Therefore, the main course for changes and differences of bacterial community composition between invaded and uninvaded soil may be due to the selective assembly of the bacterial community [47]. Presumably, invasion by *B*. *alba* tends to alter the community structure of bacteria rather than diversity, thereby facilitating further invasion processes [48].

Anthropogenic activities have been recorded to alter land-use patterns [49], and the degree of human disturbance on soil has been recorded to gradually increase from farmland to forest. Bacterial communities in forest soil therefore often exhibit a different structure between invaded and uninvaded soil. For example, results from our investigation indicated that *B*. *alba* invasion reduced the abundance of Alphaproteobacteria in forest soil and increased its abundance in other land-use types. This result may be due to Alphaproteobacteria undergoing weak soil perturbation and providing habitats rich in organic matter.

Evidence from previous investigations indicates that plant invasion can result in a significant change in the structure and function of the native ecosystem [50–52]. Invasion of *B*. *alba* is associated with changes in the soil environment of coastal zones, including pH and EC, predominantly due to selective absorption of N forms associated to a strong preference for ammonium (NH^4+^) over nitrate (NO^3-^) and alkaline substances in litter and root exudates of invasion plants [53, 54]. Obvious changes in pH and EC may interactively influence phylogenetic diversity associating with bacterial community functions [55].

Based on RDA, soil pH and EC were the most important soil physicochemical properties accounting for bacterial community changes. As differences in soil EC among the five land-use types could potentially cause distinct shifts in the carbon source utilization patterns of soil microorganisms [56], therefore soil AK may be a main factor influencing community composition in our study.

The influence of spatial distance on microbial assemblages plays an important role in biogeographic research. Variation partitioning analysis quantified the contribution of spatial distance and environmental variables to bacterial community variation. A high proportion of the variation of bacterial communities can therefore be explained by a combination of environmental variables, indicating that environmental selection is a major process in coastal microbial assemblages. In addition, environmental variables solely contribute more to variation than spatial variables (PCNM variables), suggesting that heterogeneous habitat of the different land-use types plays an important role in structuring bacterial communities in the coastal zone. The identification of ten phyla that were mainly influenced by environmental selection verifies that environmental selection has a greater effect than distance. As there is no single biotic or abiotic factor that does not change in determining the composition of the soil bacteria, *B*. *alba* relative abundance may therefore influence the bacterial community [57]. With growth and dynamics of above-ground plants also being affected by land-use pattern [58], the impact of plants in our study may also be masked by interactions between soil properties, land-use type and spatial distance. As previously mentioned, Firmicutes has a high rate of dispersal and soil colonization, a factor that may be associated with the high enrichment of invasion plants [59].

In addition to bacterial diversity and community composition shifts, bacterial community network structure and their associations with environmental properties were also altered by the invasion of *B*. *alba*. Overall, numbers of total nodes and total links were higher in uninvaded soil, as well as network connectivity and average cluster coefficients. Our results suggest that, compared to microorganisms from invaded soil (total nodes 260, total links 431), microorganisms in uninvaded coastal soil (total nodes 313, total links 1148) have stronger relationships with each other and higher influences amongst networks. During invasion, interactions between bacteria members will decrease, this being contrary to our second hypothesis. This conclusion indicates that more bacteria members were functionally interacting in uninvaded soil [60]. Within soil modules, a small number of module hubs (i.e. nodes highly connected within a module) and connectors (i.e. nodes linking different modules together) were identified. In our study, Acidobacteriales may function as a keystone taxa for *B*. *alba* invasion, having a positive effect on total phosphorus. Beta-diversity changes in the forest and orchard land-use types may be associated with the south subtropical region being largely limited by phosphorus [61].

Interactions between soil physicochemical properties and land-use explain the higher variance for Acidobacteria and Nitrospirae than other phyla (Fig 4). Generalists in the network could link with both members within their own module and those belonging to other modules [29]. The results in our study show that high percentage of super generalists (Acidobacteriales) and 50% generalists belongs to Acidobacteria, which appears mostly in magnetite and quartz as Yang et al. recorded [62]. In addition, Acidobacteria could decompose litter as soil saprophytes, affecting nitrogen cycling and remineralization [63]. Nitrospirae bacteria could also oxidize ammonium and nitrite under aerobic conditions. Kong et al. and Zhang et al. found obvious change of abundance of Acidobacteria and Nitrospirae when alien plants invasion [50, 64]. Thus, in the invaded soil community in our study, *B*. *alba* may selectively assemble special keystone taxa to form a more ordered, stable and efficient community than in native soil.

## Conclusion

By examining the effects of *B*. *alba* invasion along the coastline in southern China, our results indicated that, although *B*. *alba* did not cause a change of alpha diversity of soil bacteria, it did cause a significant change in beta diversity in forest and waterfront land-use types. This finding indicates that there land-use types may be seriously affected due to plant invasion. Furthermore, the combination of occurrence network analysis, land-use type and soil prosperities may drive keystone taxa in *B*. *alba* invasion. By consideration of dynamic changes in the network, our understanding of invasion processes will be advanced.

## Supporting information

S1 Fig The rarefaction curve revealed the relationships between sample size and OTU numbers. (DOCX)

S2 Fig Variation partitioning of bacterial communities by environmental variables. (DOCX)

S3 Fig Bacterial network, left: Invaded soil, right: Uninvaded soil. (DOCX)
