# Correction: The effects of *Bidens alba* invasion on soil bacterial communities across different coastal ecosystem land-use types in southern China

**DOI:** 10.1371/journal.pone.0253358

**Published:** 2021-06-14

**Authors:** Yue Wang, Juyu Lian, Hao Shen, Yunlong Ni, Ruyun Zhang, Yun Guo, Wanhui Ye

The fourth author, Yunlong Ni, is incorrectly noted as the corresponding author. The correct corresponding author is Juyu Lian. Juyu Lian’s email address is: lianjy@scbg.ac.cn.
