# Supplementary figures and images for: The effects of Bidens alba invasion on soil bacterial communities across different coastal ecosystem land-use types in southern China
Source: PLoS One. 2020 Oct 28;15(10):e0238478. doi: 10.1371/journal.pone.0238478 (PMC7592744; doi:10.1371/journal.pone.0238478)

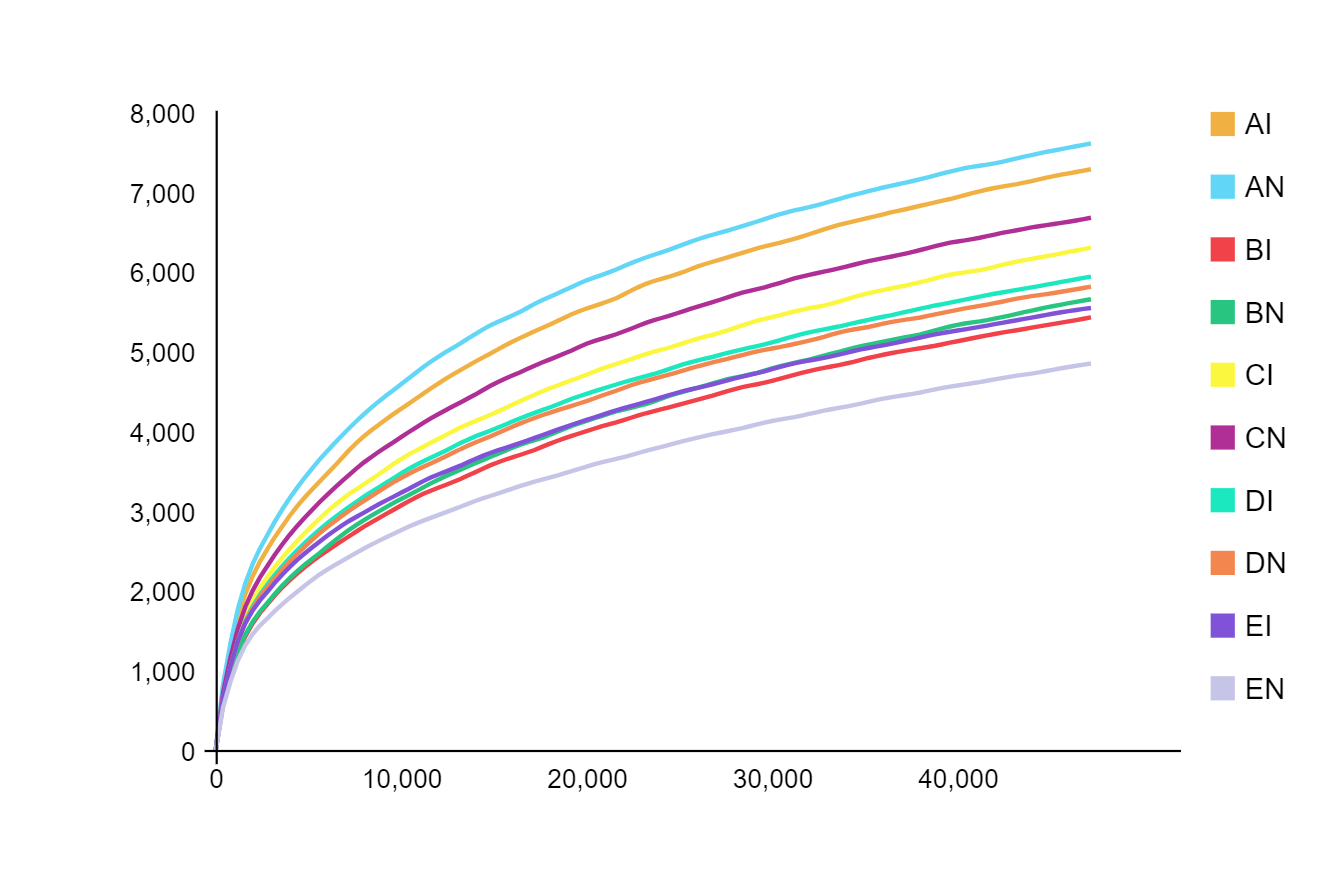
S1 Fig. The rarefaction curve revealed the relationships between sample size and OTU numbers.

Supplement: S1 Fig — (DOCX) [file pone.0238478.s001.docx]

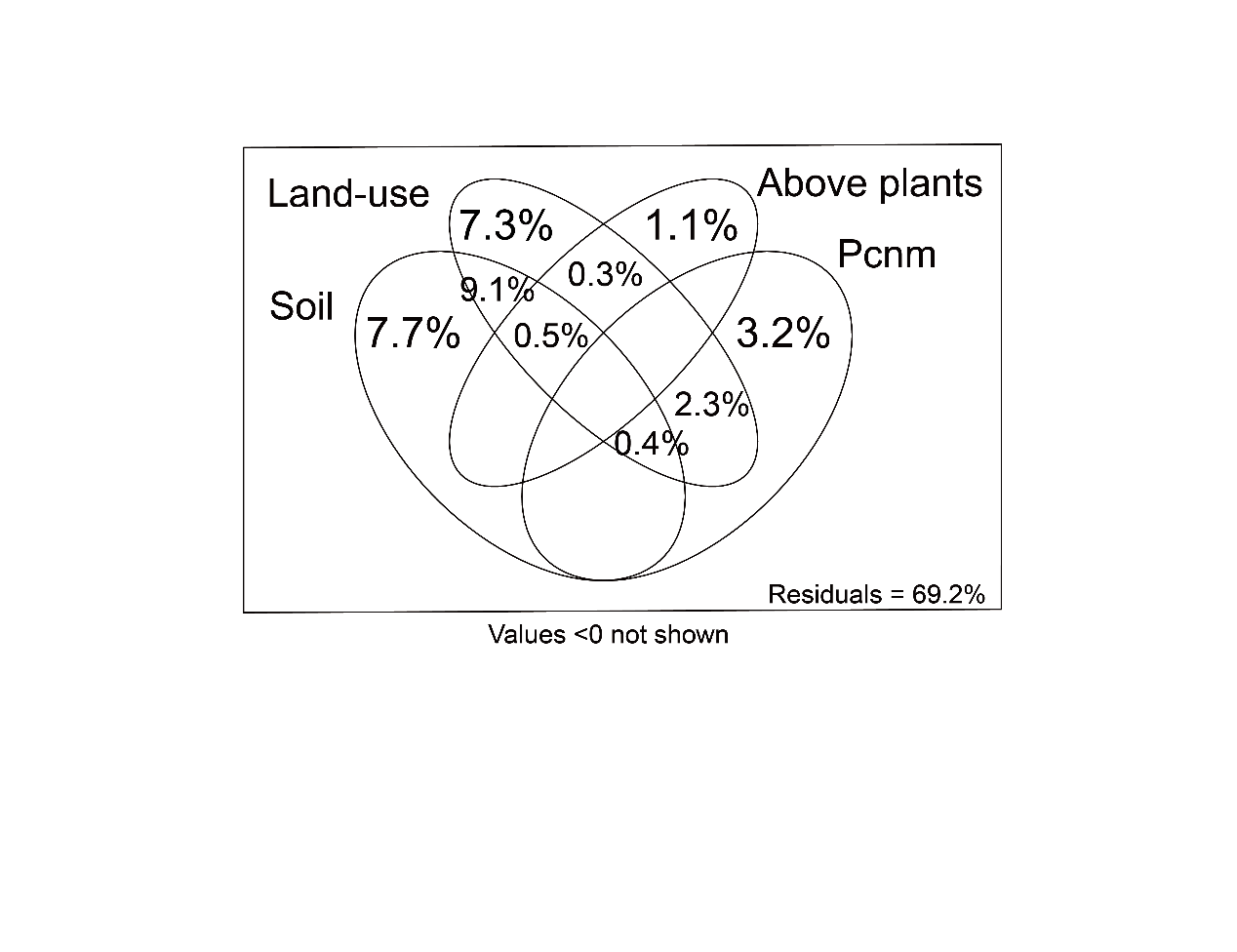


S2 Fig. Variation partitioning of bacterial communities by environmental variables.

Supplement: S2 Fig — (DOCX) [file pone.0238478.s002.docx]

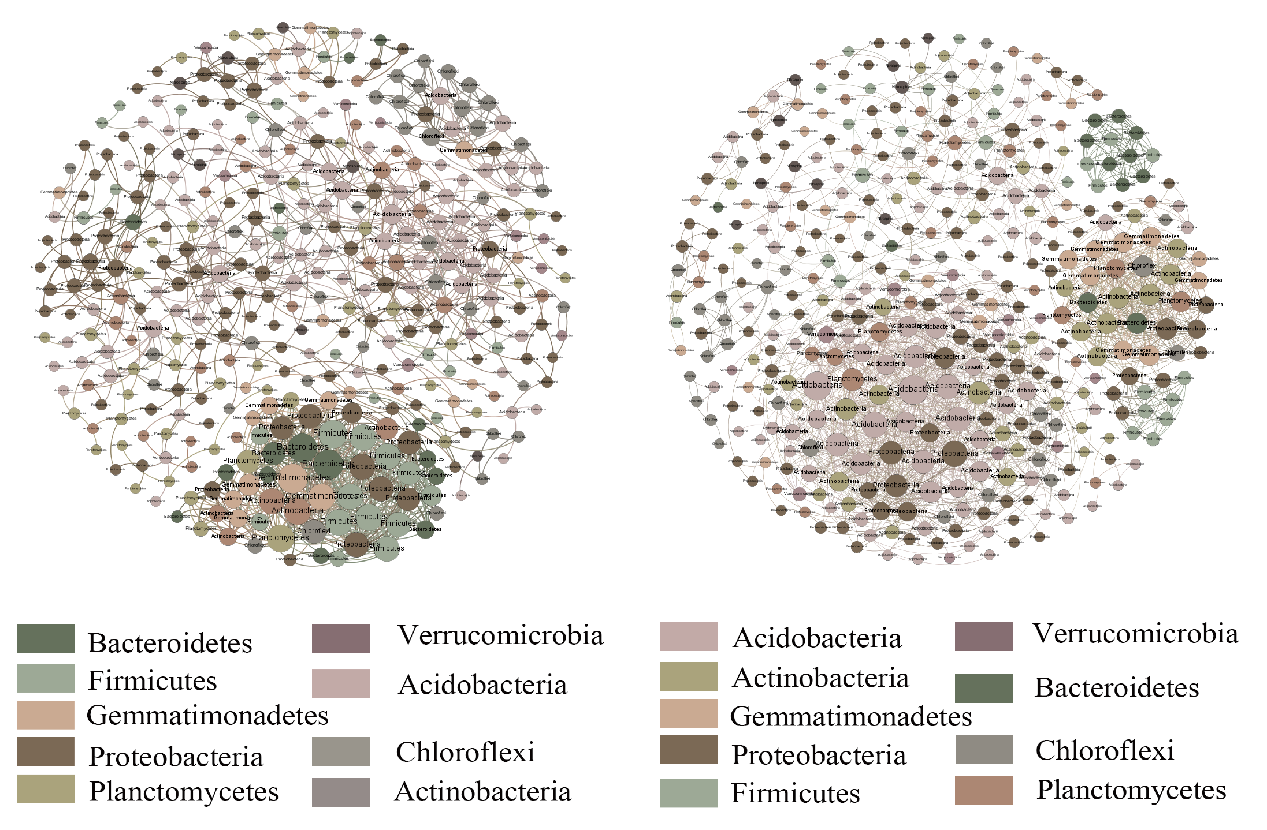


S3 Fig. Bacterial network, left: invaded soil, right: uninvaded soil

Supplement: S3 Fig — (DOCX) [file pone.0238478.s003.docx]
